# Insights into the Structural Requirements of Potent Brassinosteroids as Vegetable Growth Promoters Using Second-Internode Elongation as Biological Activity: CoMFA and CoMSIA Studies

**DOI:** 10.3390/ijms18122734

**Published:** 2017-12-17

**Authors:** Karoll Ferrer-Pertuz, Luis Espinoza, Jaime Mella

**Affiliations:** 1 Departamento de Química, Universidad Técnica Federico Santa María, Av. España No. 1680, Valparaíso 2340000, Chile; karoll.ferrer.14@sansano.usm.cl; 2 Instituto de Química y Bioquímica, Facultad de Ciencias, Universidad de Valparaíso, Casilla 5030, Avda. Gran Bretaña 1111, Playa Ancha, Valparaíso 2360102, Chile; 3 Centro de Investigación Farmacopea Chilena (CIFAR), Universidad de Valparaíso, Casilla 5030, Avda. Gran Bretaña 1111, Playa Ancha, Valparaíso 2360102, Chile

**Keywords:** brassinosteroids, bean second-internode, 3D-QSAR, CoMFA, CoMSIA, plant steroids, vegetable growth promoters

## Abstract

In the present study, we have employed the ligand-based drug design technique, 3D-QSAR, through a comparative molecular field analysis (CoMFA) and a comparative molecular similarity indices analysis (CoMSIA) to determine the key factors for the plant growth promoting activity of brassinosteroids reported in literature, using the bean second-internode bioassay measured on two groups of compounds with different molar concentrations. This is the first 3D-QSAR study using the second internode elongation as biological activity. These results provide useful ideas for the design of new molecules, which could be explored in the future to identify novel vegetable growth promoters with similar or greater biological activity than natural brassinosteroids. The reliability of this study was supported by the robust statistical parameters obtained from CoMFA (Model A, r^2^_pred_ = 0.751; Model B, r^2^_pred_ = 0.770) and CoMSIA (Model A, r^2^_pred_ = 0.946; Model B, r^2^_pred_ = 0.923) analysis.

## 1. Introduction

**Figure 1:**
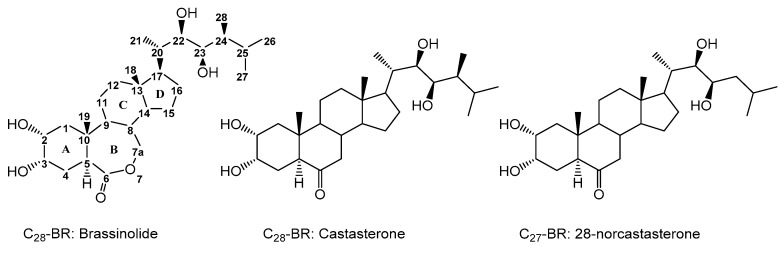
Brassinolide and other important natural occurring brassinosteroids.

Brassinosteroids (BRs) represent a group of polyhydroxylated plant steroid hormones that regulate plant growth and differentiation throughout their life cycle [1], and also mediate the environmental responses in plants [2]. These steroids include more than 70 structurally and functionally related compounds [3] with a common 5α-cholestane skeleton, which have been found at very low concentrations in all organs from a wide range of higher and lower plant species, with C28-BRs (i.e., castasterone (CS), brassinolide (BL)) and C27-BRs (i.e., 28-norcasthasterone) being the most abundant and extensively present in nature [4] (Figure 1). Up to now, 65 free brassinosteroid and 5 brassinosteroid conjugates have been detected and characterized [5].

Further work has demonstrated that BRs have a promising potential use in agriculture, since they do not only induce stem elongation, but also improve biomass formation and total crop yield, being a type of non-toxic and environmentally friendly hormone [6,7]. Moreover, BRs are recognized for their ability to stimulate growth in plants subjected to even unsuitable conditions, such as low and high temperature, excess heavy metals, salinity, water stress, drought, herbicidal injury, and pathogen attack [5,8,9], thus playing a significant role in helping the plant to overcome environmental stress.

BRs have attracted considerable interest because of the notable biological effects from their exogenous application at lower than micromolar concentrations. However, the low abundance of BRs in plant sources, and their costly and difficult synthesis have stimulated several workers to search for more accessible and bioactive analogues [10,11]. 

The most commonly used tests for evaluating the bioactivity of brassinosteroids are the bean second-internode bioassay (BSIB) and the rice lamina inclination test (RLIT) [12]. Although different studies have recognized some essential structural characteristics for high bioactivity in brassinosteroids, subsequent research has revealed that the relative activities of brassinosteroids vary in some extent with the type of bioassay used [13], and with the dosage range in a given type of assay [14], results are not always comparable, thus, comparison of data must be taken with caution.

Some attempts to understand the structure–activity relationships (SAR) of brassinosteroids have been done by several groups. The first qualitative SAR was performed by Takatsuto et al. in a series of 21 brassinosteroids [15]. Brosa et al. performed a 2D-QSAR and a Grid map study in a small series of 15 compounds [16,17]. The same group explored the SAR through calculation of the molecular electrostatic potential of the compounds [18,19]. From the discovery of the crystal structure of the BR receptor [20,21], studies with docking technique have been performed [3]. Despite these efforts, it should be noted that, to this date, a study based on 3D-QSAR analysis using the bean second-internode bioassay is not available yet. In this sense, the aim of our study is related to generating a model with high ability for predicting the activity of new analogs and providing suggestions for the design of new potent plant growth regulators with the best synthetic cost–bioactivity relationship in order to improve the benefits of these hormones in agriculture. A comparative molecular field analysis (CoMFA) and Comparative Molecular Similarity Indices Analysis (CoMSIA) approach on two groups of brassinosteroids using bean second-internode bioassay at different concentrations has been employed to achieve this aim.

## 2. Results

### 2.1. Statistical Results of Comparative Molecular Field Analysis (CoMFA) and Comparative Molecular Similarity Indices Analysis (CoMSIA)

**Figure 2:**
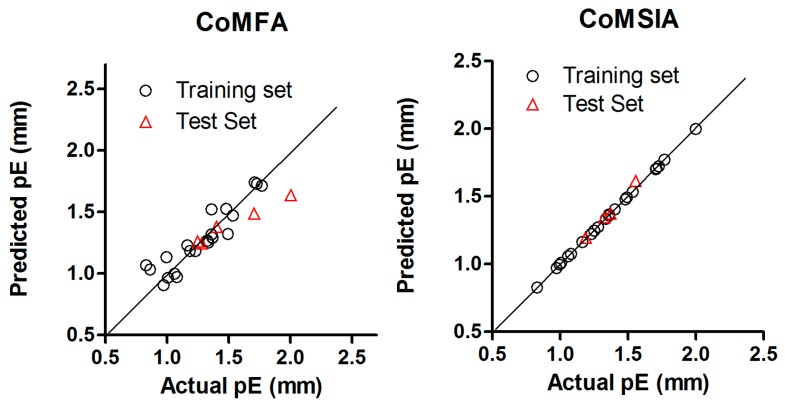
Scatter plots of actual versus predicted pE values by CoMFA (left) and CoMSIA (right) analysis for Model A.

**Figure 3:**
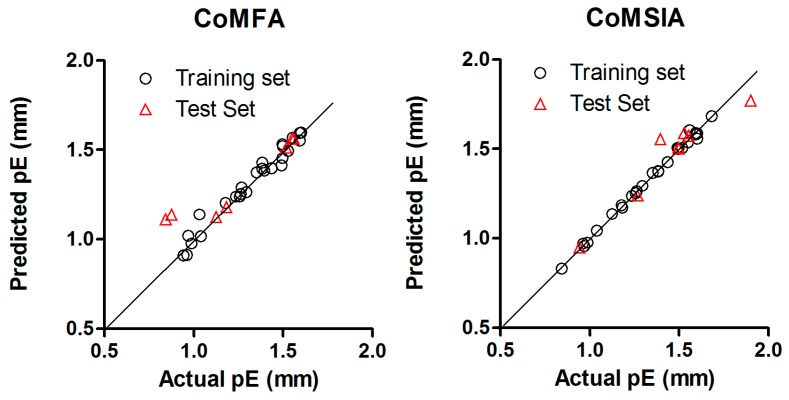
Scatter plots of actual versus predicted pE values by CoMFA (left) and CoMSIA (right) analysis for Model B.

The statistical parameters supporting all possible field combinations of CoMFA and CoMSIA models were listed in Table 1 and Table 2, respectively. The best models were used to predict the plant growth promoting activity for the BRs of the test set, the scattered plots between actual and predicted values for the best CoMFA and CoMSIA models of both training set and test set are shown in Figure 2 and Figure 3. In addition, Table 3 and Table 4 show the biological activity (pE values) for training and test set. Residual values are also reported for each compound. All the residual values were found below ±0.4 within a tolerable error range.

#### 2.1.1. CoMFA Statistics

The analysis of the obtained 3D-QSAR models (Table 1 and Table 2) showed that the best CoMFA models are not always obtained using a combination of electrostatic and steric fields. Model A, using electrostatic field, produced a cross-validated q^2^ of 0.622 with an optimum number of components *N* = 2 and a non-cross-validated r_ncv_^2^ value of 0.860. Model B, using both steric and electrostatic fields, gave a cross validated q^2^ value of 0.810 with an optimum number of components *N* = 3, a non-cross-validated r_ncv_^2^ of 0.968, an estimated F-value of 221.25, and low standard error of estimation (SEE) value of 0.041, while the contribution of steric and electrostatic fields was 28.2% and 71.8%, respectively.

#### 2.1.2. CoMSIA Statistics

Unlike CoMFA, CoMSIA has the advantage that it generates more information. The fields obtained by CoMSIA are steric (S), electrostatic (E), hydrophobic (H), hydrogen-bond donor (D), and hydrogen-bond acceptor (A) fields. Thirty-one different CoMSIA models were developed using various combinations of CoMSIA descriptor fields (Table 1 and Table 2). The satisfactory q^2^, r_ncv_^2^, and r^2^_pred_ values of the models were the most important selection criterion for the selection of the best CoMSIA model. In Model A, the best field contribution was CoMSIA-EA, which has a good cross-validated correlation coefficient q^2^ value of 0.723 with an optimum number of components *N* = 7, a significant r_ncv_^2^ of 1.000, lower SEE of 0.004, and higher F-value of 11,379.460. The electrostatic and hydrogen-bond acceptor descriptors had 49.1% and 50.9% of relative contributions. On the other hand, in Model B, the best combination of descriptors was CoMSIA-EHA, that was built using eight components and has cross-validated q^2^ value of 0.719, while the non-cross-validated r_ncv_^2^ was 0.996, with a low SEE of 0.019 and an estimated high F-value of 492.026. Electrostatic, hydrophobic, and hydrogen-bond acceptor contributions were found to be 39.2%, 55.7%, 27.4, and 33.4%, respectively. 

### 2.2. Validation of the 3D-QSAR Models

The best CoMFA and CoMSIA models were generated employing PLS analysis, which produced the cross-validated coefficients q^2^. A 3D-QSAR model should possess a high q^2^ value, but this is not the only condition that a model must exhibit in order to have an adequate predictive capacity [22]. For this purpose, we carried out the external validation of the 3D-QSAR models. The external validation was carried out by setting aside a test set of compounds not included in the construction of the model. The r^2^_pred_ values of the CoMFA models, A and B, were found to be 0.751 and 0.770, while the $r_{m}^{2}$ had a value of 0.561 and 0.640 for models A and B, respectively. On the other hand, our results indicate that CoMSIA models were able to describe the test set variance with a high predictability for both models. This is demonstrated by the high r^2^_pred_ values obtained for all models (Model A, r^2^_pred_ = 0.946, $r_{m}^{2}$ = 0.875; Model B, r^2^_pred_ = 0.923, $r_{m}^{2}$ = 0.880).

The CoMFA model B together with the CoMSIA models A and B passed Tropsha’s recommended test for predictive ability, unlike the CoMFA model A, whose ${r^{\prime }}_{0}^{2}$ did not come close to value of $r^{2}$ and $(r^{2}-{r^{\prime }}_{0}^{2})/r^{2}$ value was >0.1. The statistical parameters of the predictability of the best CoMFA and CoMSIA analysis, and the acceptability criteria of the QSAR models are represented in the Table 5 and Table 6.

### 2.3. 3D-QSAR Contour Maps

**Figure 4:**
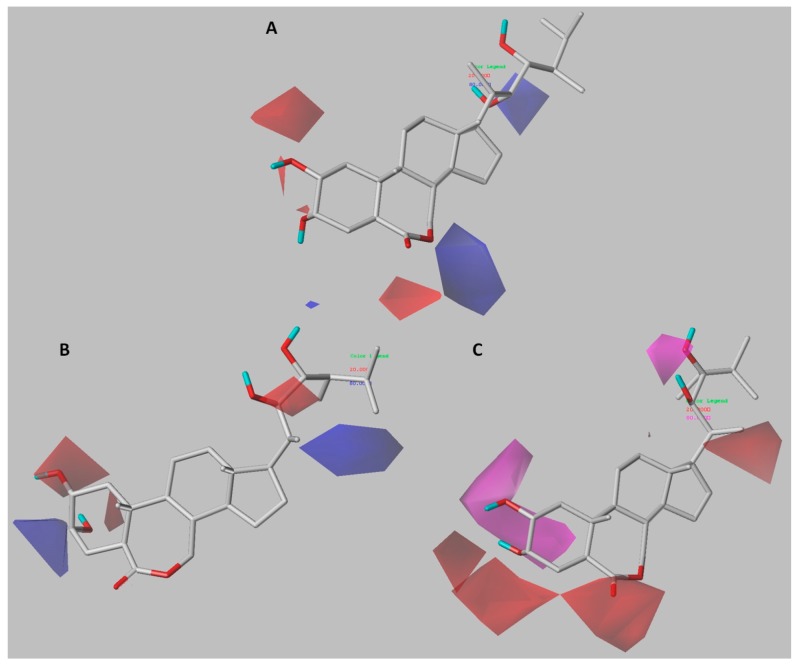
CoMFA and CoMSIA contour maps of Model A around the most active compound **6a**. CoMFA contour maps: (**A**) electrostatic field distribution, electronegative (red) and electropositive (blue) favorable fields; CoMSIA contour maps: (**B**) electrostatic field contribution, the colors have the same meaning as in CoMFA contour maps, and (**C**) hydrogen-bond acceptor field contribution, favorable (magenta) and unfavorable (red).

**Figure 5:**
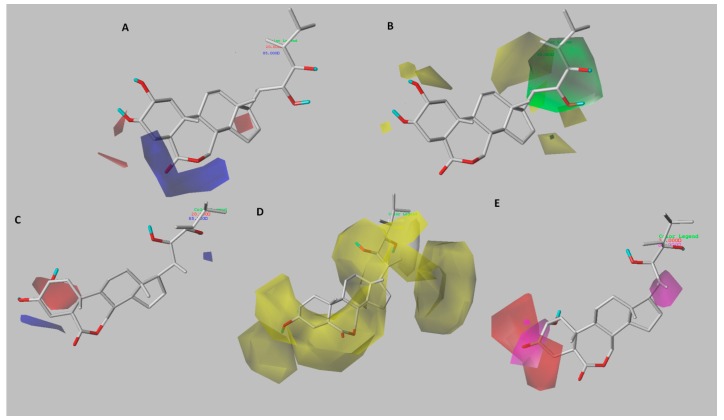
CoMFA and CoMSIA contour maps for brassinosteroids ligands of the Model B. Compound **6a** (most active of the series) is shown inside the fields. CoMFA contour maps: (**A**) electrostatic field contribution, electropositive (blue) and electronegative (red) favorable fields, and (**B**) steric field contribution, favorable (green) and unfavorable (yellow); CoMSIA contour maps: (**C**) electrostatic field distribution, the colors have the same meaning as in CoMFA contour maps, (**D**) hydrophobic field distribution, favorable (yellow) and unfavorable (white), and (**E**) hydrogen-bond acceptor field contribution, favorable (magenta) and unfavorable (red).

Unlike a 2D-QSAR equation, the results of a 3D-QSAR study can be viewed graphically. The color contour maps obtained show the regions of the molecule where structural modifications can be made. With this information, it is possible to propose changes in the steric, electrostatic, hydrophobic, and hydrogen bonding properties of the studied compounds. With the structure–activity relationship thus obtained, it is possible to rationally design new brassinosteroids with promising biological activity. The contour maps obtained from CoMFA and CoMSIA models, along with template compound, are shown in Figure 4 and Figure 5. Compound **6a**, the most active of the series, has been selected as the reference structure in each presented map.

## 3. Discussion

### 3.1. Analysis of CoMFA Contour Maps

#### 3.1.1. Model A

Figure 4a depicts the distribution of electrostatic field using compound **6a** as a reference structure. The blue and red (80% and 20% contributions) contour maps represent favorable electropositive charge areas and favorable electronegative charge areas, respectively. A big blue polyhedron is present near the 7th position of the brassinolide **6a** (on the top part of the lactone ring), which suggests that electron-donating groups at this position would be favorable. This can be proven experimentally by considering the low activity of the compounds **2a**, **4a**, **5a**, and **8a**, which direct the 7-oxo group at the B-ring toward the blue contour, unlike compounds with the carbonyl group oriented under the plane that show a higher activity (**6a**, **18a** and **21a**). Therefore, a pattern of type 7-oxolactone in B-ring would be less favorable, which is consistent with previous reports indicating that type 6-oxolactone brassinosteroid analogs are more active [15]. Moreover, the spatial orientation of the carbonyl group is influenced by the position of the hydroxyl substituents on the A-ring. The compounds **7a** and **9a** have a 2,3-dihydroxy substitution pattern, and they project the carbonyl group under the plane that is favorable for the activity, while the homologues **8a** and **10a** have a 3,4-dihydroxy substitution pattern that disfavors the B-ring conformation, causing the carbonyl group to be projected toward blue contour. Two red regions are sighted in the proximity of the α-oriented hydroxyl groups at C-2 and C-3 positions. This suggests that electronegative substitutions are favorable for growth-promoting activity (e.g., compounds **3a**, **7a**, **13a**, **11a**, and **21a**), whereas compounds with the β-oriented hydroxyl groups at C-2 and C-3 positions showed lower activity (e.g., compounds **22a**, **23a**, **24a**, **26a**, and **27a**). The α-oriented hydroxyl group at A-ring has been reported to be essential for greater biological activity [5].

#### 3.1.2. Model B

Figure 5a,b show the distribution of steric and electrostatic fields around compound **6a**. In the steric contour map (80% and 20% contributions), green polyhedrons show regions where the increase in volume improves activity. While yellow polyhedrons indicate that the increase in volume is unfavorable for activity. Two yellow regions are found around the hydroxyl group at C-2 position, suggesting that the presence of bulky groups at this position disfavor the activity. The presence of these yellow isopleths supports previous reports regarding the presence of the hydroxyl group at 2-α position as not indispensable to elicit the biological activity [23]. This can be explained by the fact that compounds such as **2b**, **3b**, **12b**, **10b**, **7b**, **18b**, **13b**, and **20b** exhibited comparable activity values, with respect to those that did have 2-α substitution (e.g., compounds **4b**, **5b**, **21b**, **23b**, **6a**, **21a**). Furthermore, the presence of a large green contour around the side chain, that is flanked by yellow isopleths, would allow a limited increase in volume in this region, in order to improve the biological activity of BRs. In fact, a group of compounds referred to as ‘‘superbrassinolides’’ has been described, that had superior activity to brassinolide. This series of derivatives contained chains of varying length and cycloalkyl groups with different ring sizes at C-24, reaching the conclusion that the bioactivity clearly increased inversely with the chain length [14]. Therefore, excessive chain elongation prevents adequate binding to the receptor.

The map of electrostatic contours (85% and 20% contributions) shows red and blue polyhedrons. Red polyhedrons show areas where the presence of electronegative atoms improves biological activity. While blue polyhedrons show areas where electro-positive atoms are favorable for biological activity. One big blue contour surrounding the positions C-4 to C-8 of the A and B-rings indicates that the electropositive potential favors the activity. Therefore, the presence of a polar functional group from the B-ring is not essential for biological activity (e.g., **12a**), which appears to be in strong contrast with the structure requirements mentioned in previous papers [24]. This information is consistent with the electrostatic field contour map for CoMFA-model A.

### 3.2. Analysis of CoMSIA Contour Maps

#### 3.2.1. Model A

The contour plots for CoMSIA-EA are presented in Figure 4b,c ,which illustrates the electrostatic and hydrogen-bond acceptor fields using compound **6a** as reference structure. All contour maps were generated with 80% and 20% contributions for favorable and unfavorable interactions, respectively.

The electrostatic field effect is shown in Figure 4b, two red isopleths are sighted in the α-hydroxyls at C-2 and C-3 positions, suggesting that electron-withdrawing groups are favorable to increase activity, as it was also observed in the electrostatic contour map for CoMFA-model A. One blue contour around the β-position at C-3 shows that the presence of electronegative groups in the α-position generates charge deficiency on either the carbon or hydrogen atom in the same position, which is favorable for biological activity. A red contour around the hydroxyl group at C-22 position of the brassinolide side chain suggests that this group is more important for the activity than the hydroxyl group at C-23 position. This is in accordance with previous reports, which consider that having a 22,23-vicinal diol in the side chain of a compound is not absolutely necessary to exhibit typical brassinosteroid activity [25], a requirement that had previously been established as a key structural feature [26,27]. The presence of a blue contour near the carbon atoms in the side chain of fluorinated compounds (**15a** and **18a**) indicates that use of electronegative groups is better than use of electropositive groups. However, some studies have shown activity in analogues without substituents on the chain, with shorter side chains or even analogues without side chain [28,29,30,31,32].

Figure 4c shows the distribution of hydrogen-bond acceptor field. Magenta and red contour maps represent favorable and unfavorable positions for hydrogen-bond acceptor groups. Two magenta isopleths around the hydroxyl groups at C-2 and C-23 positions suggest that the presence of hydrogen-bond acceptor group at those positions might enhance the activity. On the other hand, two red contours are sighted in the proximity of the hydroxyl group at C-3 position, which suggests that it would be more appropriate to functionalize with H-bond acceptor groups. This is consistent with the blue isopleth at the same position in the electrostatic contour map. Another two red isopleths were found in the hydrogen-bond acceptor contour map, one surrounding the carbonyl group at B-ring and the other in the hydroxyl group at C-20 position indicating that H-bond acceptor group at this position may be unfavorable. Compounds **25a** and **26a**, which contain an OH group at C-20 position are less active, while compounds hydroxylated at C-17 position (**16a** and **21a**) do not have this restriction, and have better activity.

#### 3.2.2. Model B

Figure 5c–e present the CoMSIA-EHA contour maps that illustrates the electrostatic, hydrophobic, and hydrogen-bond acceptor fields around compound **6a** as reference structure. 

As shown in Figure 5c, CoMSIA electrostatic (blue favored 85%, and red disfavored 20%) contour map was comparatively similar to the electrostatic contour maps of CoMFA model A and CoMSIA model A. Since this field was already explained, this will not be explained here again. The hydrophobic field effect is shown in Figure 5d, the presence of the yellow and white color (80% and 20% contributions) contour maps explain the favorable and unfavorable influence of the hydrophobic fields, respectively. A big yellow contour is sighted with clear predominance throughout the structure showing projections towards the side chain and at the 3rd position of the brassinolide (**6a**). Therefore, these positions appear to be optimal for modulating lipophilicity of the compounds indicating that hydrophobic groups at those positions would be favorable. This information is consistent with reports that BRs are lipophilic compounds, characterized by a 5α-cholestane skeleton, oxygenated at least at C-3, C-22 and C-23 [33].

Figure 5e shows the contour map for hydrogen-bond acceptor field (magenta-favored 80% and red disfavored 20%). A magenta isopleth around the α-oriented hydroxyl groups at C-2 and C-3 positions, and one big red contour surrounding the 3rd position suggest that possible polyhydroxylations in A-ring would be favorable, with an alpha substitution pattern in both positions, as already was discussed while describing the CoMFA electrostatic contour map for model A. Another magenta contour appeared at C-17 position, indicating that substituents containing hydrogen-bond acceptor group directly attached to D-ring might enhance the activity. This can be explained by the fact that compounds having hydroxyl directly attached at C-17 are among the most active of the series (**16b**, **18b**, **20b**, **21b**, **25b**, **26b**, and **21a**).

#### 3.2.3. SAR Summary

**Figure 6:**
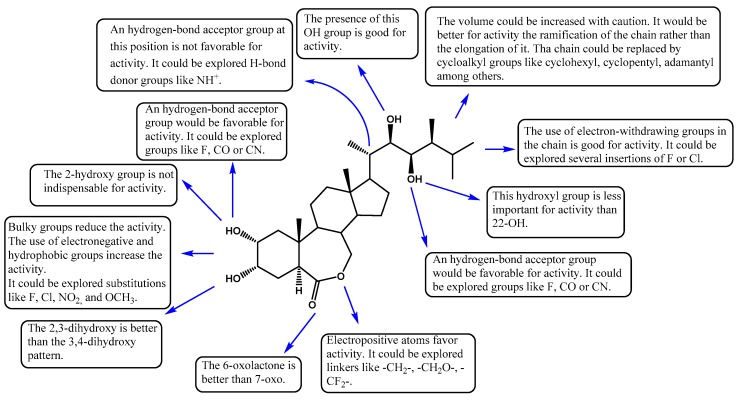
Summary of the main SAR discussed and found in this study.

In order to systematize the main structural—activity relationships found and discussed in this study, Figure 6 presents the main modifications that can be explored on the brassinosteroid system. The areas that can be modified are in rings A, B, and in the chain at position 17.

## 4. Materials and Methods

### 4.1. Data Sets Selection and Biological Activity

A set of 27 molecules with biological activity tested at 10^−9^ M (Model A) and a set of 38 molecules with biological activity tested at 10^−10^ M (Model B) were selected from available literature [3,28,29,30,34,35]. In both models, the bean second-internode bioassay was used as biological activity for the generation of the 3D-QSAR models (CoMFA and CoMSIA). The biological activity of the data set compounds was reported as E value (elongation, in millimeters), which spanned across a wide range 2.50–38.50 mm for Model A and 0.80–54.80 mm for Model B. The E values were converted into pE values using the formula log(100 × E/Emax). The training and test sets were randomly chosen in 8:2 ratio. Both sets contain compounds with varied biological activities and various structural modifications. This allows guaranteeing an adequate predictive capacity of the models. The chemical structures of all molecules, along with their E and pE values, are shown in Table 7 and Table 8.

### 4.2. Molecular Alignment

The entire study was carried out in the Sybyl X software [36]. Each structure was previously minimized by the Tripos force field (1000 iterations) [37]. The term gradient was adjusted to 0.005 Kcal/mol·Å. The calculation of atomic charges for each structure was carried out using the Gaisteiger-Hückel method [38]. Each minimized structure was then subjected to simulated annealing dynamics. The structures were heated to 1000 K per 1000 femtoseconds (fs), and then cooled to 50 K per 1000 fs. The best final conformers were selected for the construction of the final CoMFA and CoMSIA models. The database thus obtained was aligned using distill rigid alignment protocol.

### 4.3. CoMFA and CoMSIA Field Calculation

The aligned database was positioned in the center of a cubic lattice with a grid spacing of 2 Å. A carbon atom with charge +1 and an atomic radius of 1.52 Å was used as a probe to calculate the potentials. The energy cut-off value was set by default at 30 Kcal/mol. To reduce noise and speed up the calculation of potentials, the column filtering value was set to 2.0 Kcal/mol. In the case of CoMSIA, for the calculation of hydrophobic and hydrogen-bond potentials, a probe atom with hydrophobicity +1 and HBD/HBA of +1 was used. The attenuation factor α was set by default at 0.3 [39].

### 4.4. Internal Validation and Partial Least Squares (PLS) Analysis

The search for a correlation between biological activity (dependent variable) and calculated potentials (independent variables) for CoMFA and CoMSIA was carried out by means of PLS statistical analysis. Regression analysis was performed through leave-one-out (LOO) cross-validation procedure using SAMPLS method [40].

In CoMFA and CoMSIA, the cross-validation analysis was applied to determine the value of the cross-validation coefficient (q^2^), the cross-validated standard error of predictions (SEP), and the optimal number of components (N). The q^2^ value is a measure of the internal quality of the models which was calculated using the following formula:

(1) $$q^{2}=1-\frac{\sum {\left(y_{i}-y_{\mathrm{pred}}\right)}^{2}}{\sum {\left(y_{i}-\bar{y}\right)}^{2}}$$

where $y_{i}$, $\bar{y}$, and $y_{\mathrm{pred}}$ are the observed, mean, and predicted activity in the training set, respectively.

Final non-cross validated conventional analysis [41] was generated with the optimal number of components equal to that yielding the highest q^2^, and the corresponding conventional correlation coefficient r_ncv_^2^ was obtained.

In addition, the statistical significance of the models was described by its standard error of estimate (SEE) and the probability value (*F*-value).

### 4.5. 3D-QSAR External Validation

The external predictive capacity of each built model was evaluated by calculating the predictive correlation coefficient (r^2^_pred_) [42,43], which was obtained from the following equation:

(2) $$r_{\mathrm{pred}}^{2}=\frac{\mathrm{SD}-\mathrm{PRESS}}{\mathrm{SD}}$$

where SD is the sum of squared deviations between the biological activities of the test set molecules, and the mean activities of the training molecules and PRESS is the sum of squared deviations between actual and predicted activity values for each molecule in the test set [44,45]. For a predictive QSAR model, the value of r^2^_pred_ should be more than 0.6.

Moreover, the models were also subjected to external validation criteria according to the proposed test by Golbraikh and Tropsha [22]. The external predictive power of the developed QSAR models using the test set was examined by considering $r_{m}^{2}$ metrics, as shown below [46]:

(3) $$r_{m}^{2}=r^{2}\left(1-\sqrt{r^{2}-r_{0}^{2}}\right)$$

where $r^{2}$ and $r_{0}^{2}$ are squared correlation coefficients between the observed and predicted activities of the test set with and without intercept, respectively. For a significant external model validation, the value of $r_{m}^{2}$ should be more than 0.5.

Tropsha et al. [47] considered a QSAR model predictive, if the following conditions are satisfied:

(4) $$q^{2}>0.5$$

(5) $$r^{2}>0.6$$

(6) $$\frac{\left(r^{2}-r_{0}^{2}\right)}{r^{2}}<0.1\;\mathrm{or}\;\frac{\left(r^{2}-{r^{\prime }}_{0}^{2}\right)}{r^{2}}<0.1$$

(7) $$0.85\leq k\leq 1.15\;\mathrm{or}\;0.85\leq k^{\prime }\leq 1.15$$

It has been demonstrated [22] that all of the above criteria are indeed necessary to adequately assess the predictive ability of a QSAR model.

## 5. Conclusions

Brassinosteroid analogues previously reported in literature have been studied by using 3D-QSAR analysis. CoMFA and CoMSIA approaches were carried out to determine structural requirements for improving potency of brassinosteroid analogues as plant-growth promoters using the bean second-internode bioassay. Overall, the statistical results of both models studied at different molar concentrations exhibited good correlation, good predictive power and satisfactory agreement with previous literature reports. The 3D contour maps showed that the growth promoting activity of the compounds was influenced mainly by electrostatic properties and the presence of hydrogen-bond acceptor groups. The information obtained in this study provides useful suggestions that can be used in the successful design, development and synthesis of novel derivatives. The actual synthesis of new derivatives is on-going and will later be screened for its biological activity.

## Figures and Tables

**Table 1:** Summary of results from comparative molecular field analysis (CoMFA) and Comparative Molecular Similarity Indices Analysis (CoMSIA) analysis for Model A ^a^.

Models	q^2^	N	SEP	SEE	r_ncv_^2^	F	Relative % Contributions				
							S	E	H	D	A
CoMFA-S	−0.396	1	0.336	0.183	0.584	26.690	1	-	-	-	-
CoMFA-E	0.622	2	0.180	0.109	0.860	55.213	-	1	-	-	-
CoMFA-SE	0.607	2	0.183	0.09	0.904	84.813	35.6	64.4	-	-	-
CoMSIA-S	−0.164	10	0.479	0.057	0.982	59.415	1	-	-	-	-
CoMSIA-E	0.570	12	0.305	0.009	1.000	1876.640	-	1	-	-	-
CoMSIA-H	0.286	3	0.278	0.098	0.912	61.914	-	-	1	-	-
CoMSIA-D	0.326	3	0.270	0.150	0.792	22.83	-	-	-	1	-
CoMSIA-A	0.649	4	0.200	0.063	0.965	117.696	-	-	-	-	1
CoMSIA-SE	0.596	12	0.296	0.004	1.000	12,519.923	23.0	77.0	-	-	-
CoMSIA-SEH	0.573	13	0.323	0.002	1.000	55,261.759	11.7	59.4	29.0	-	-
CoMSIA-SEHD	0.581	6	0.233	0.010	0.999	3325.404	7.5	35.3	19.1	38.1	-
CoMSIA-SEHA	0.639	8	0.233	0.011	0.999	1953.948	7.1	37.8	17.3	-	37.9
CoMSIA-SED	0.589	6	0.231	0.013	0.999	1782.188	11.0	45.0	-	44.0	-
CoMSIA-SEA	0.697	10	0.232	0.006	1.000	5084.953	10.2	44.6	-	-	45.2
CoMSIA-SEDA	0.662	7	0.217	0.010	0.999	2619.974	6.3	33.3		31.9	28.5
CoMSIA-SH	0.253	3	0.284	0.095	0.917	66.043	23.1	-	76.9	-	-
CoMSIA-SD	0.255	2	0.276	0.156	0.761	30.321	14.9	-	-	85.1	-
CoMSIA-SA	0.576	4	0.220	0.058	0.971	142.753	16.4	-	-	-	83.6
CoMSIA-SHD	0.462	11	0.324	0.001	1.000	89,668.574	10.9	-	39.3	49.8	-
CoMSIA-SHA	0.536	4	0.231	0.049	0.979	202.281	10.2	-	32.1	-	57.7
CoMSIA-SDA	0.490	4	0.242	0.071	0.956	92.436	9.5	-	-	40.1	50.4
CoMSIA-SHDA	0.514	12	0.324	0.001	1.000	87,368.769	6.6	-	26.5	35.7	31.2
CoMSIA-EH	0.602	13	0.311	0.002	1.000	60,678.767	-	63.8	36.2	-	-
CoMSIA-ED	0.601	6	0.228	0.019	0.997	876.360	-	49.8	-	50.2	-
CoMSIA-EA	0.723	7	0.239	0.004	1.000	11,379.460	-	49.1	-	-	50.9
CoMSIA-EHD	0.598	6	0.229	0.011	0.999	2455.905	-	37.3	21.7	41.0	-
CoMSIA-EHA	0.660	5	0.204	0.022	0.996	794.462	-	37.6	21.2	-	41.2
CoMSIA-EDA	0.682	7	0.210	0.011	0.999	2511.362	-	34.6	-	34.5	30.9
CoMSIA-EHDA	0.647	6	0.214	0.013	0.999	2074.207	-	28.5	15.1	30.7	25.7
CoMSIA-HD	0.516	11	0.307	0.001	1.000	92,707.250	-	-	45.5	54.5	-
CoMSIA-HA	0.571	4	0.222	0.049	0.979	196.681	-	-	36.9	-	63.1
CoMSIA-HDA	0.555	9	0.269	0.007	1.000	4120.755	-	-	28.5	37.9	33.6
CoMSIA-DA	0.559	12	0.309	0.042	0.992	93.531	-	-	-	46.0	54.0
CoMSIA-ALL	0.631	6	0.219	0.013	0.999	2002.943	5.0	27.7	13.9	29.1	24.3

^a^ N is the optimal number of components, q^2^ is the square of the LOO cross-validation (CV) coefficient, SEP is the standard error of prediction, r_ncv_^2^ is the square of the non-CV coefficient, SEE is the standard error of estimation of non-CV analysis, F is the *F*-test value. S, E, H, D, and A are the steric, electrostatic, hydrophobic, hydrogen-bond donor and hydrogen-bond acceptor fields contribution, the best selected CoMFA and CoMSIA models are highlighted in bold character.

**Table 2:** Summary of results from CoMFA and CoMSIA analysis for Model B ^a^.

Models	q^2^	N	SEP	SEE	r_ncv_^2^	F	Relative % Contributions				
							S	E	H	D	A
CoMFA-S	−0.114	2	0.239	0.116	0.739	32.512	1	-	-	-	-
CoMFA-E	0.803	2	0.100	0.060	0.930	152.542	-	1	-	-	-
CoMFA-SE	0.810	3	0.101	0.041	0.968	221.25	28.2	71.8	-	-	-
CoMSIA-S	0.285	1	0.276	0.225	0.145	4.082	1	-	-	-	-
CoMSIA-E	0.585	3	0.164	0.091	0.872	49.948	-	1	-	-	-
CoMSIA-H	0.367	3	0.203	0.104	0.833	36.509	-	-	1	-	-
CoMSIA-D	0.200	2	0.223	0.161	0.584	16.162	-	-	-	1	-
CoMSIA-A	0.339	3	0.207	0.123	0.767	24.199	-	-	-	-	1
CoMSIA-SE	0.618	3	0.158	0.076	0.91	74.262	22.6	77.4	-	-	-
CoMSIA-SEH	0.604	3	0.160	0.067	0.932	100.153	13.8	57.7	28.5	-	-
CoMSIA-SEHD	0.710	8	0.156	0.017	0.996	601.957	9.3	34.0	21.7	35.0	-
CoMSIA-SEHA	0.711	10	0.166	0.012	0.998	985.125	9.6	35.3	22.6	-	32.5
CoMSIA-SED	0.628	3	0.155	0.068	0.929	96.290	14.8	45.6	-	39.6	-
CoMSIA-SEA	0.657	3	0.149	0.069	0.927	92.442	14.7	45.8	-	-	39.5
CoMSIA-SEDA	0.609	3	0.159	0.074	0.915	79.125	11.1	34.1	-	31.0	23.8
CoMSIA-SH	0.269	3	0.218	0.107	0.824	34.261	23.5	-	76.5	-	-
CoMSIA-SD	0.413	9	0.229	0.026	0.992	231.079	32.7	-	-	67.3	-
CoMSIA-SA	0.548	20	0.359	0.003	1.000	7159.795	25.0	-	-	-	75.0
CoMSIA-SHD	0.633	5	0.162	0.047	0.970	127.937	15.2	-	36.2	48.7	-
CoMSIA-SHA	0.69	19	0.272	0.001	1.000	86,018.515	15.5	-	33.9	-	50.7
CoMSIA-SDA	0.458	5	0.197	0.051	0.964	107.301	19.1	-	-	42.6	38.3
CoMSIA-SHDA	0.639	5	0.161	0.044	0.973	146.386	11.4	-	25.7	35.7	27.3
CoMSIA-EH	0.624	14	0.221	0.004	1.000	5870.861	-	61.1	38.9	-	-
CoMSIA-ED	0.579	3	0.165	0.086	0.886	56.746	-	53.5	-	46.5	-
CoMSIA-EA	0.599	3	0.161	0.086	0.886	56.816	-	54.1	-	-	45.9
CoMSIA-EHD	0.705	8	0.157	0.019	0.996	513.854	-	37.4	26.9	35.7	-
CoMSIA-EHA	0.719	8	0.154	0.019	0.996	492.026	-	39.2	27.4	-	33.4
CoMSIA-EDA	0.562	3	0.169	0.090	0.876	51.739	-	38.3	-	35.3	26.5
CoMSIA-EHDA	0.686	8	0.162	0.019	0.996	512.390	-	29.7	22.9	27.9	19.5
CoMSIA-HD	0.625	5	0.164	0.051	0.963	104.326	-	-	47.4	52.6	-
CoMSIA-HA	0.666	19	0.282	0.001	1.000	14,7123.304	-	-	44.3	-	55.7
CoMSIA-HDA	0.621	5	0.165	0.049	0.966	114.981	-	-	32.4	38.9	28.8
CoMSIA-DA	0.245	2	0.217	0.143	0.669	23.220	-	-	-	57.0	43.0
CoMSIA-ALL	0.7	9	0.164	0.014	0.998	755.591	8.1	27.4	18.1	27.0	19.5

^a^ N is the optimal number of components, q^2^ is the square of the LOO cross-validation (CV) coefficient, SEP is the standard error of prediction, r_ncv_^2^ is the square of the non-CV coefficient, SEE is the standard error of estimation of non-CV analysis, F is the *F*-test value. S, E, H, D and A are the steric, electrostatic, hydrophobic, hydrogen-bond donor and hydrogen-bond acceptor fields contribution, the best selected CoMFA and CoMSIA models are highlighted in bold character.

**Table 3:** Actual and predicted pE values of molecules in the Model A generated through the best CoMFA and CoMSIA analysis ^a^.

Molecule	Actual pE (mm)	CoMFA		CoMSIA	
		Predicted pE (mm)	Residual	Predicted pE (mm)	Residual
**1a**	1.5318	1.4708	0.06	1.5338	0.00
**2a**	1.0056	0.9666	0.04	1.0086	0.00
**3a**	1.4790	1.5260	−0.05	1.4780	0.00
**4a**	1.3336	1.2516	0.08	1.3326	0.00
**5a**	1.0580	0.9970	0.06	1.0570	0.00
**6a** ^t^	2.0000	1.6380	0.36	1.9990	0.00
**7a** ^t,u^	1.5544	1.4410	0.11	1.6120	−0.06
**8a** ^u^	0.8617	1.0327	−0.17	1.1680	−0.31
**9a**	1.7667	1.7127	0.05	1.7687	0.00
**10a** ^u^	1.3688	1.2908	0.08	1.3730	0.00
**11a**	1.7263	1.7293	0.00	1.7243	0.00
**12a** ^u^	0.8125	1.4180	−0.61	1.3220	−0.51
**13a**	1.4901	1.3231	0.17	1.4941	0.00
**14a** ^t^	1.2470	1.2600	−0.01	1.2474	0.00
**15a**	0.9943	1.1333	−0.14	0.9953	0.00
**16a**	1.3590	1.5220	−0.16	1.3670	−0.01
**17a**	1.2275	1.1835	0.04	1.2245	0.00
**18a** ^t^	1.7046	1.4880	0.22	1.6996	0.01
**19a** ^u^	1.3230	1.2650	0.06	1.3420	−0.02
**20a**	1.3590	1.3170	0.04	1.3588	0.00
**21a**	1.7068	1.7398	−0.03	1.7058	0.00
**22a** ^u^	1.1854	1.1804	0.01	1.1940	−0.01
**23a** ^t^	1.4013	1.3800	0.02	1.4033	0.00
**24a**	1.0773	0.9713	0.11	1.0753	0.00
**25a**	0.8295	1.0695	−0.24	0.8265	0.00
**26a**	0.9708	0.9068	0.06	0.9706	0.00
**27a** ^t^	1.2779	1.2430	0.03	1.2749	0.00

^a^ CoMFA-E, and CoMSIA-EA, 10^−9^ M assay. ^t^ test set compounds used in CoMFA, ^u^ test set compounds used in CoMSIA.

**Table 4:** Actual and predicted pE values of molecules in the Model B generated through the best CoMFA and CoMSIA analysis ^a^.

Molecule	Actual pE (mm)	CoMFA		CoMSIA	
		Predicted pE (mm)	Residual	Predicted pE (mm)	Residual
**1b**	1.4942	1.4542	0.04	1.4992	−0.01
**2b** ^u^	1.2655	1.2905	−0.03	1.2400	0.03
**3b** ^t^	1.1245	1.1260	0.00	1.1385	−0.01
**4b**	1.2946	1.2646	0.03	1.2956	0.00
**5b**	1.0394	1.0184	0.02	1.0454	−0.01
**6b**	0.9688	1.0188	−0.05	0.9578	0.01
**7b**	1.0321	1.1411	−0.11	1.4260	−0.39
**8b** ^t^	0.8410	1.1134	−0.27	0.8320	0.01
**9b**	0.1643	1.0641	−0.90	1.1240	−0.96
**10b**	1.4344	1.3964	0.04	1.4284	0.01
**11b**	1.2343	1.2393	−0.01	1.2373	0.00
**12b**	1.2569	1.2409	0.02	1.2539	0.00
**13b**	1.3818	1.4298	−0.05	1.3778	0.00
**14b**	1.3818	1.3958	−0.01	1.3738	0.01
**15b** ^u^	1.4942	1.5332	−0.04	1.5100	−0.02
**16b**	1.4891	1.4141	0.08	1.5071	−0.02
**17b** ^t^	1.5579	1.5627	0.00	1.6059	−0.05
**18b** ^u^	1.5260	1.4940	0.03	1.5890	−0.06
**19b**	1.5916	1.5956	0.00	1.5826	0.01
**20b** ^u^	1.8987	1.5444	0.35	1.7690	0.13
**21b** ^u^	1.5513	1.5693	−0.02	1.5760	−0.02
**22b**	1.5997	1.5977	0.00	1.5577	0.04
**23b**	1.3511	1.3751	−0.02	1.3671	−0.02
**24b**	1.5916	1.5526	0.04	1.5896	0.00
**25b** ^u^	1.3948	1.3858	0.01	1.5560	−0.16
**26b**	1.5997	1.3252	0.27	1.5887	0.01
**6a**	2.0000	1.4404	0.56	1.5060	0.49
**8a** ^t^	1.5490	1.5644	−0.02	1.5380	0.01
**10a** ^u^	1.4967	1.5217	−0.03	1.5030	−0.01
**12a** ^t^	1.5165	1.5136	0.00	1.5045	0.01
**15a**	1.2612	1.2562	0.01	1.2652	0.00
**21a**	1.6812	1.3303	0.35	1.6852	0.00
**22a**	1.1750	1.2050	−0.03	1.1850	−0.01
**23a** ^u^	0.9425	0.9105	0.03	0.9510	−0.01
**24a** ^t^	1.1803	1.1804	0.00	1.1733	0.01
**25a** ^t^	0.8740	1.1388	−0.26	1.1280	−0.25
**26a**	0.9602	0.9132	0.05	0.9732	−0.01
**27a**	0.9855	0.9785	0.01	0.9785	0.01

^a^ CoMFA-SE, and CoMSIA-EHA, 10^−10^ M assay. ^t^ test set compounds used in CoMFA, ^u^ test set compounds used in CoMSIA.

**Table 5:** Statistical parameters of the predictability of the best CoMFA and CoMSIA analysis ^a^.

	SD	PRESS	r^2^_pred_
**10^−9^ M**			
**CoMFA-E**	0.7219	0.1798	0.751
**CoMSIA-EA**	0.0699	0.0038	0.946
**10^−10^ M**			
**CoMFA-SE**	0.6282	0.1446	0.770
**CoMSIA-EHA**	0.6269	0.0484	0.923

^a^ SD is the sum of the squared deviations between the biological activity of molecules in the test set and mean activity of the training set molecules, PRESS is the sum of the squared deviations between predicted and actual biological activity values for every molecule in the test set, r^2^_pred_ is the predictive correlation coefficient based only on the test set molecules.

**Table 6:** External validation characteristics of different models according to Golbraikh and Tropsha [22].

Parameters	Threshold Value	Test Results			
		Model A		Model B	
		CoMFA	CoMSIA	CoMFA	CoMSIA
$q^{2}$	>0.5	0.622	0.723	0.810	0.719
$r^{2}$	>0.6	974	0.994	0.884	0.911
${r^{\prime }}_{0}^{2}$	Close to value of $r^{2}$	0.794	0.980	0.808	0.909
$k^{\prime }$	0.85 < *k*′ < 1.15	1.098	0.983	0.949	0.990
$(r^{2}-{r^{\prime }}_{0}^{2})/r^{2}$	<0.1	0.185	0.014	0.086	0.001
$r_{m}^{2}$	>0.5	0.561	0.875	0.640	0.880

**Table 7:** Chemical structures of brassinosteroid analogues of Model A (10^−9^ M) with their actual activity.

No.	Compound	Elongation (mm)	pE
1a	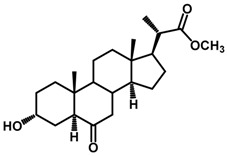	13.10	1.5318
2a	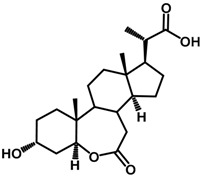	3.90	1.0056
3a	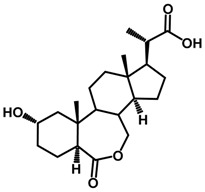	11.60	1.4790
4a	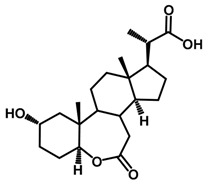	8.30	1.3336
5a	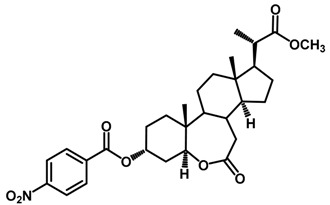	4.40	1.0580
6a	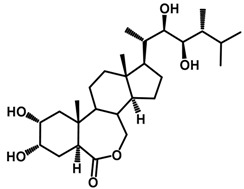	38.50	2.0000
7a	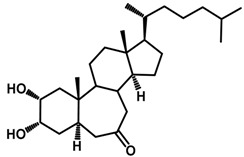	13.80	1.5544
8a	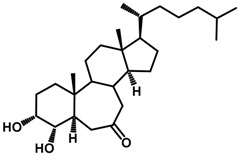	2.80	0.8617
9a	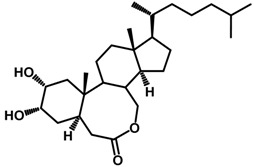	22.50	1.7667
10a	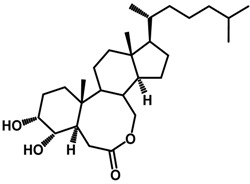	9.00	1.3688
11a	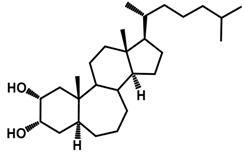	20.50	1.7263
12a	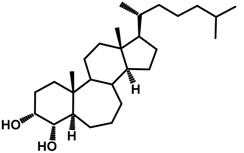	2.50	0.8125
13a	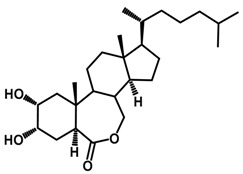	11.90	1.4901
14a	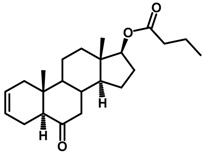	6.80	1.2470
15a	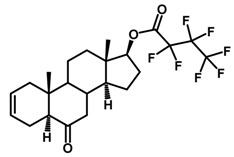	3.80	0.9943
16a	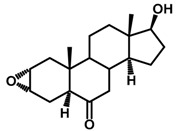	8.80	1.3590
17a	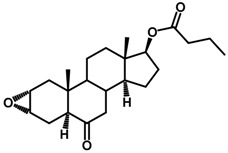	6.50	1.2275
18a	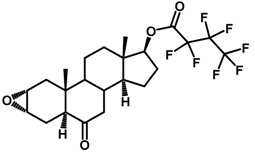	19.50	1.7046
19a	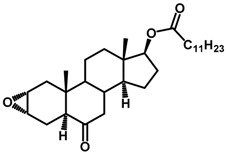	8.10	1.3230
20a	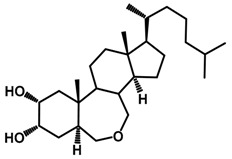	8.80	1.3590
21a	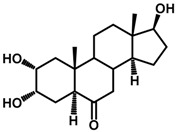	19.60	1.7068
22a	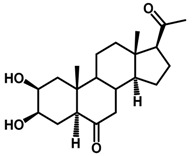	5.90	1.1854
23a	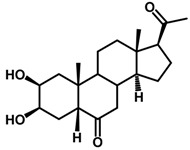	9.70	1.4013
24a	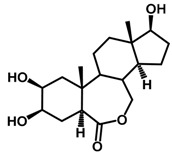	4.60	1.0773
25a	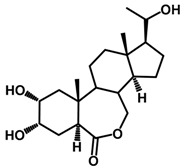	2.60	0.8295
26a	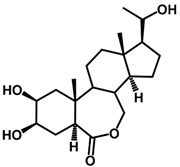	3.60	0.9708
27a	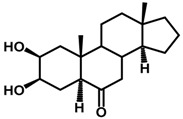	7.30	1.2779

**Table 8:** Chemical structures of brassinosteroid analogues of model B (10^−10^ M) with their actual activity.

No.	Compound	Elongation (mm)	pE
1b	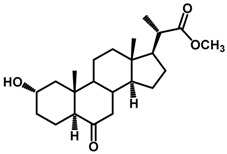	17.10	1.4942
2b	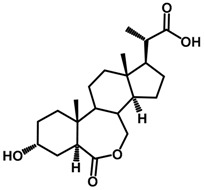	10.10	1.2655
3b	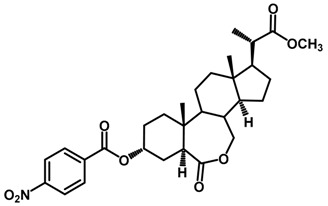	7.30	1.1245
4b	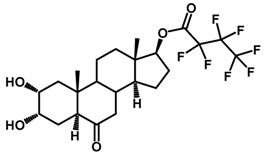	10.80	1.2946
5b	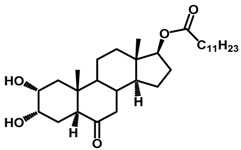	6.00	1.0394
6b	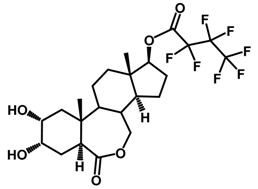	5.10	0.9688
7b	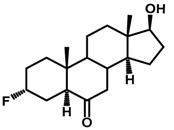	5.90	1.0321
8b	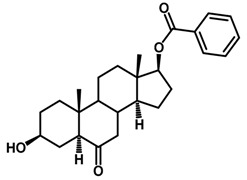	3.80	0.8410
9b	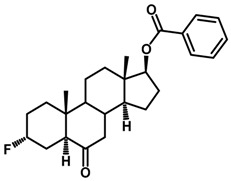	0.80	0.1643
10b	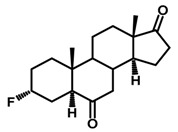	14.90	1.4344
11b	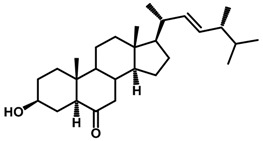	9.40	1.2343
12b	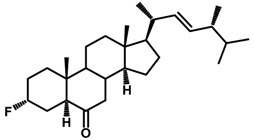	9.90	1.2569
13b	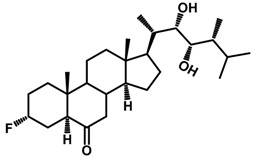	13.20	1.3818
14b	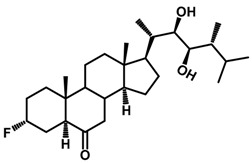	13.20	1.3818
15b	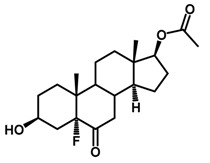	17.10	1.4942
16b	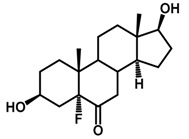	16.90	1.4891
17b	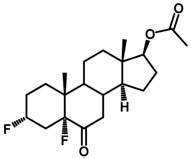	19.80	1.5579
18b	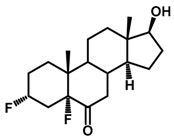	18.40	1.5260
19b	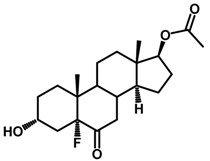	21.40	1.5916
20b	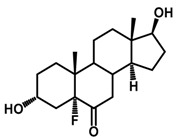	43.40	1.8987
21b	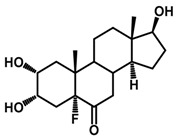	19.50	1.5513
22b	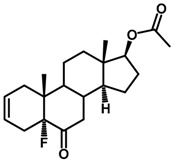	21.80	1.5997
23b	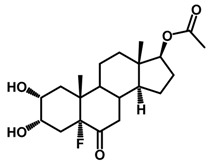	12.30	1.3511
24b	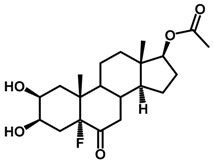	21.40	1.5916
25b	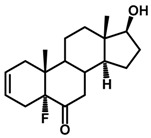	13.60	1.3948
26b	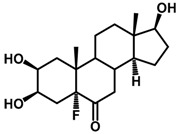	21.80	1.5997
6a	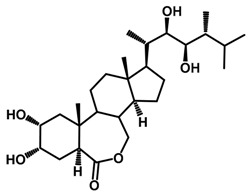	54.80	2.0000
8a	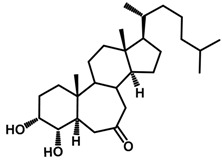	19.40	1.5490
10a	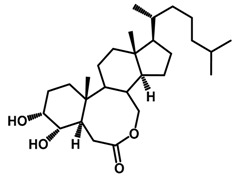	17.20	1.4967
12a	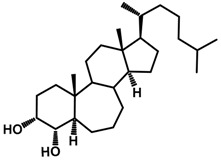	18.00	1.5165
15a	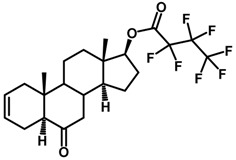	10.00	1.2612
21a	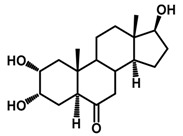	26.30	1.6812
22a	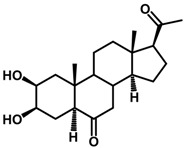	8.20	1.1750
23a	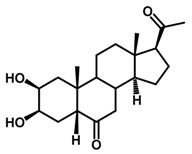	4.80	0.9425
24a	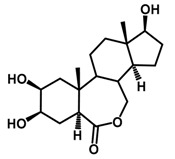	8.30	1.1803
25a	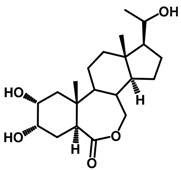	4.10	0.8740
26a	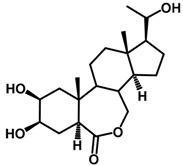	5.00	0.9602
27a	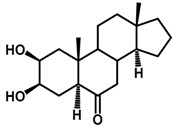	5.30	0.9855

## Abbreviations

3D-QSAR	Three-Dimensional Quantitative Structure–Activity Relationship
CoMFA	Comparative Molecular Field Analysis
CoMSIA	Comparative Molecular Similarity Index Analysis
PLS	Partial Least Squares
LOO	Leave-One-Out
N	Optimal Number of Components
r_ncv_^2^	Non-Cross Validation Coefficient
q^2^	Cross Validation Coefficient
r^2^_pred_	Predictive Correlation Coefficient
*F*	Fischer-Test Value
SEE	Standard Error of Estimation
SEP	Standard Error of Prediction
